# Epigenetic landscape of testis specific histone H2B variant and its influence on sperm function

**DOI:** 10.1186/s13148-021-01088-4

**Published:** 2021-05-01

**Authors:** Aniket Patankar, Rahul Gajbhiye, Suchitra Surve, Priyanka Parte

**Affiliations:** 1 Department of Gamete Immunobiology, ICMR- National Institute for Research in Reproductive Health, Parel, Mumbai, 400012 India; 2 Department of Clinical Research, ICMR- National Institute for Research in Reproductive Health, Parel, Mumbai, 400012 India

**Keywords:** TH2B, Chromatin remodeling, Sperm chromatin compaction, ChIP-seq, Testis specific Histone Variant, IVF/ICSI, Sperm function, Sperm RNA

## Abstract

**Background:**

Biological relevance of the major testis specific histone H2B variant (TH2B) in sperm is not fully understood. Studies in TH2A/TH2B double knockout male mice indicate its role in chromatin compaction and male fertility. Additionally, the presence of TH2B and TH2A reportedly generates more dynamic nucleosomes, leading to an open chromatin structure characteristic of transcriptionally active genome. Given that mature human sperm are transcriptionally and translationally inactive, the presence of TH2B in mature sperm is intriguing. To address its role in sperm, we investigated the genome-wide localization of TH2B in sperm of fertile men.

**Results:**

We have identified the genomic loci associated with TH2B in fertile human sperm by ChIP-seq analysis. Bioinformatic analysis revealed ~ 5% sperm genome and 5527 genes to be associated with TH2B. Out of these 105 (1.9%) and 144 (2.6%) genes showed direct involvement in sperm function and early embryogenesis, respectively. Chromosome wide analysis for TH2B distribution indicated its least distribution on X and Y chromosomes and varied distribution on autosomes. TH2B showed relatively higher percentage of gene association on chromosome 4, 18, 3 and 2. TH2B enrichment was more in promoter and gene body region. Gene Ontology (GO) analysis revealed signal transduction and associated kinase activity as the most enriched biological and molecular function, respectively. We also observed the enrichment of TH2B at developmentally important loci, such as HOXA and HOXD and on genes required for normal sperm function, few of which were validated by ChIP-qPCR. The relative expression of these genes was altered in particular subgroup of infertile men showing abnormal chromatin packaging. Chromatin compaction positively correlated with sperm- motility, concentration, viability and with transcript levels of PRKAG2 and CATSPER B.

**Conclusion:**

ChIP-seq analysis of TH2B revealed a putative role of TH2B in sperm function and embryo development. Altered expression of TH2B associated genes in infertile individuals with sperm chromatin compaction defects indicates involvement of TH2B in transcriptional regulation of these genes in post meiotic male germ cells. This altered transcriptome may be a consequence or cause of abnormal nuclear remodeling during spermiogenesis.

**Supplementary Information:**

The online version contains supplementary material available at 10.1186/s13148-021-01088-4.

## Introduction

Spermatogenesis is a well synchronized and tightly regulated process by which male germ cells are formed. It is broadly divided into 3 phases, namely, mitotic proliferation, meiotic division and spermiogenesis. Chromatin remodeling is a landmark event in spermiogenesis during which nucleohistone to nucleoprotamine transition takes place in male germ cells. It initiates with histone hyperacetylation followed by replacement of somatic histones with testis specific histone variants. Knockout studies with these variants testify their essential role in male infertility [1, 2]. Most of these variants are found to be involved in open chromatin structure formation. This uncondensed state of sperm genome may be related to two biological events, which occur during spermiogenesis, (1) Active transcription of genes which may be required during spermiogenesis and or early embryo development, and (2) Replacement of the variants by transition proteins and protamines leading to genome compaction.

Replacement of histones by protamines is not 100% and about 5–15% histones are retained in mature human spermatozoa [3, 4]. The repertoire of retained histones comprise of canonical as well as testis specific histone variants along with several modified histones like H3K4me3, H3K27me3, etc. Several studies have demonstrated altered chromatin compaction and increased histone retention in the sperm of infertile men [5–9]. The altered chromatin compaction in sperm has also been correlated with adverse IVF and ICSI outcomes [10, 11]. These studies suggest that nucleosomal retention in sperm is programmed and any imbalance in this retention hampers chromatin compaction and consequently fertility.

Previous sperm nucleosome mapping studies employing either comparative genome hybridization or MNase-sequencing approach, have shown the enrichment of retained nucleosomes at gene regulatory elements [4, 12, 13]. Contrary to this, Carone et al. [14] and Samans et al. [15] observed even distribution of nucleosomes within distal intergenic regions, introns, centromere repeats and retro transposons with poor occupancy at gene regulatory elements like 5′ UTR, 3′ UTR, TSS, and TTS region. These contradictory observations can be attributed to alternate bioinformatics analysis, as suggested by Royo’s group [16]. Whilst the information on genome-wide distribution is available for the histone H3 and its modified forms H3K4me3/H3K27me3 [4, 17], no such information is available for TH2B, which is the major testis specific histone variant present in mature human spermatozoa. TH2B differs from H2B mainly at its N-terminus, which in H2B has been shown to be associated with chromosome condensation in meiotic cells [18]. Nucleosomal core particles containing TH2A/TH2B reportedly show fewer histone–DNA contacts, suggesting that their presence promotes an open chromatin structure [19].

Spermatozoa of male mice lacking TH2A and TH2B show increased histone retention during spermiogenesis consequently leading to infertility in these mice [20]. However, there is very little information available on the presence of testis specific histone variants in sperm of fertile men, and their retention status in infertile men. Whether this would have an implication on the status of genes in proximity of these testis specific histones, is not known.

In the present study, we have identified genomic loci associated with TH2B in the sperm of fertile men by high-throughput sequencing, and association of a few genes was confirmed using ChIP-qPCR. qRT-PCR analysis was done to determine the relative abundance of transcripts of genes associated with TH2B in sperm from fertile- and infertile men and the observations corroborated with their chromatin compaction status.

## Results

### Specific immunoprecipitation of TH2B from fertile human sperm

**Fig. 1:**
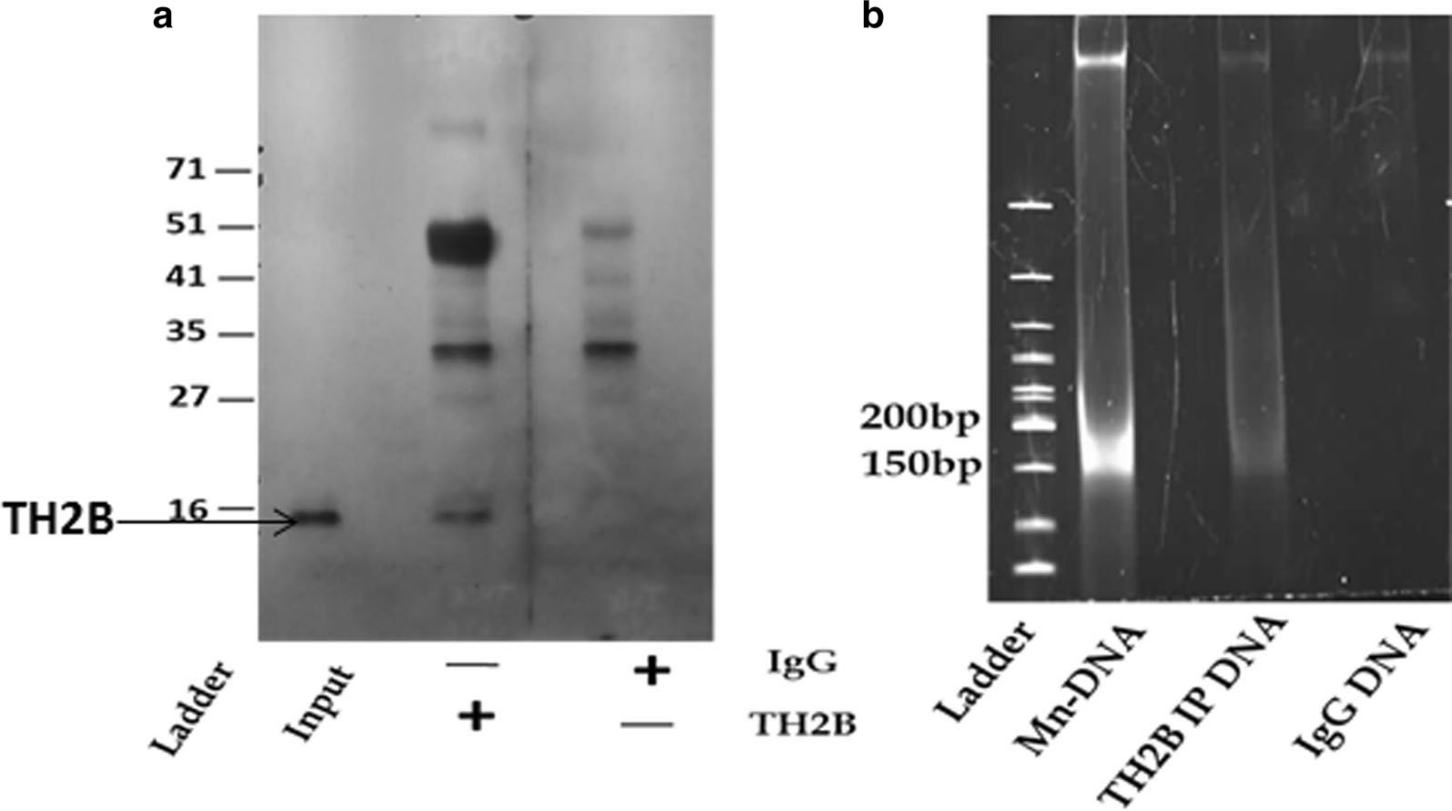
Chromatin immunoprecipitation of TH2B: ChIP-Western analysis shows immunoprecipitation of TH2B containing nucleosome specifically in TH2B pulldown but not in IgG (**a**). A representative image of one of the sample showing a band at around 146 bp on 5% polyacrylamide gel for DNA isolated from input (Mn- DNA) and ChIP-TH2B. No band was seen for DNA from ChIP-IgG (**b**)

Sperm from four healthy and proven fertile individuals were processed as described in ‘Materials and Methods’ and Chromatin immunoprecipitation (ChIP) was done to isolate TH2B bearing nucleosomes. ChIP was performed using either anti-TH2B antibody or its Isotype control (IgG). 1.2 million sperm (1/10th of the cells used for ChIP) were MNase digested, proteins were precipitated and used as positive control (Input). TH2B immunoprecipitation was confirmed by ChIP-Western analysis. ChIP-Western blot detected a band at approx. 15 kDa in immunoprecipitation product of TH2B but not of IgG, confirming specific immunoprecipitation of TH2B (Fig. 1a).

The mononucleosomal DNA from Input, TH2B-ChIP and IgG-ChIP was visualized on 5% polyacrylamide gel (Fig. 1b). The mononucleosomal DNA bands were excised, DNA was extracted out and subjected to high-throughput sequencing.

Semen parameters of the fertile individuals enrolled for the ChIP-seq analysis and values of their percent aniline blue positive and Chromomycin A3 (CMA3) positive sperm are listed in Table 1.

**Table 1:** Semen parameters and chromatin compaction status of fertile individuals enrolled for ChIP-seq

Sample No	% Progressive Motility	Concentration × 10^6^/ml	% Viability	% Aniline blue positive sperm	% CMA3 positive sperm
1	74	74	85	43	33
2	71	47.43	78	49	33
3	65	60.12	68	46	30
4	62	26.06	70	41	43

### Genome-wide distribution of TH2B and gene ontology (GO) analysis of TH2B associated genes

**Fig. 2:**
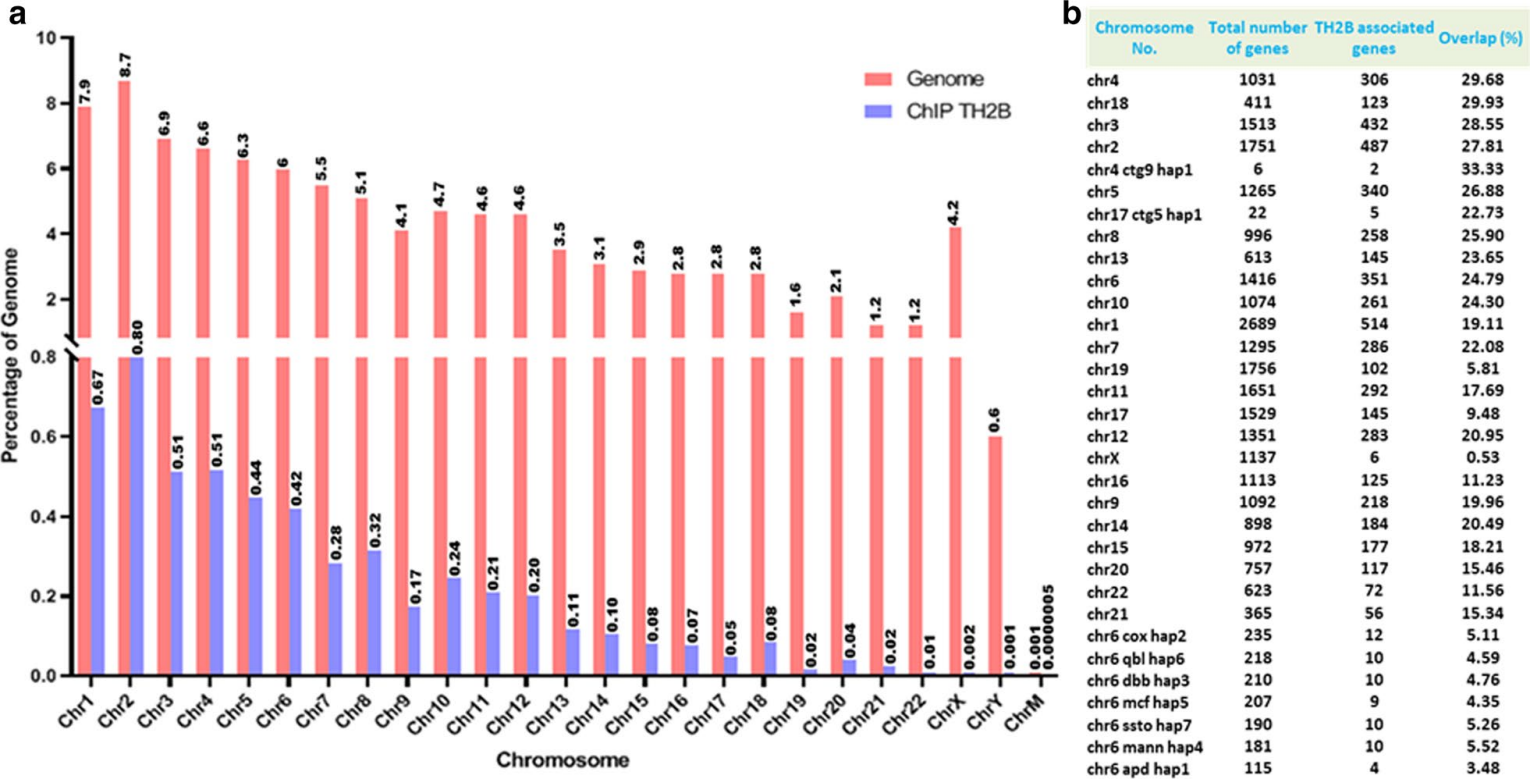
Distribution of TH2B across all chromosomes. Pink bars indicate the percentage of genome covered by individual chromosome while blue bars indicate percentage of genome occupied by TH2B with respect to entire genome (**a**). The percentage of TH2B associated genes on individual chromosomes with respect to the total number of genes on that chromosome (**b**)

Enrichment of TH2B was visualized across the human chromosomes using Cistrome tool in Galaxy genome browser [21]. TH2B distribution was noted on all autosomes in varying amount. In all, 5% of the total genome was observed to be associated with TH2B. Interestingly, sex chromosomes i.e. Chromosome X and Y showed least distribution of TH2B (Fig. 2a). When percentage of genes occupied by TH2B relative to total number of genes on a particular chromosome was considered, TH2B was found to be more enriched on genes of chromosome 4, 18, 3 and 2. In case of sex chromosome TH2B was observed to be present on regulatory RNAs (miRNAs) on X chromosome while none of the Y chromosome genes were found to be associated with TH2B (Fig. 2b). The chromosome wise distribution of TH2B associated genes is provided as Additional file 1.

**Fig. 3:**
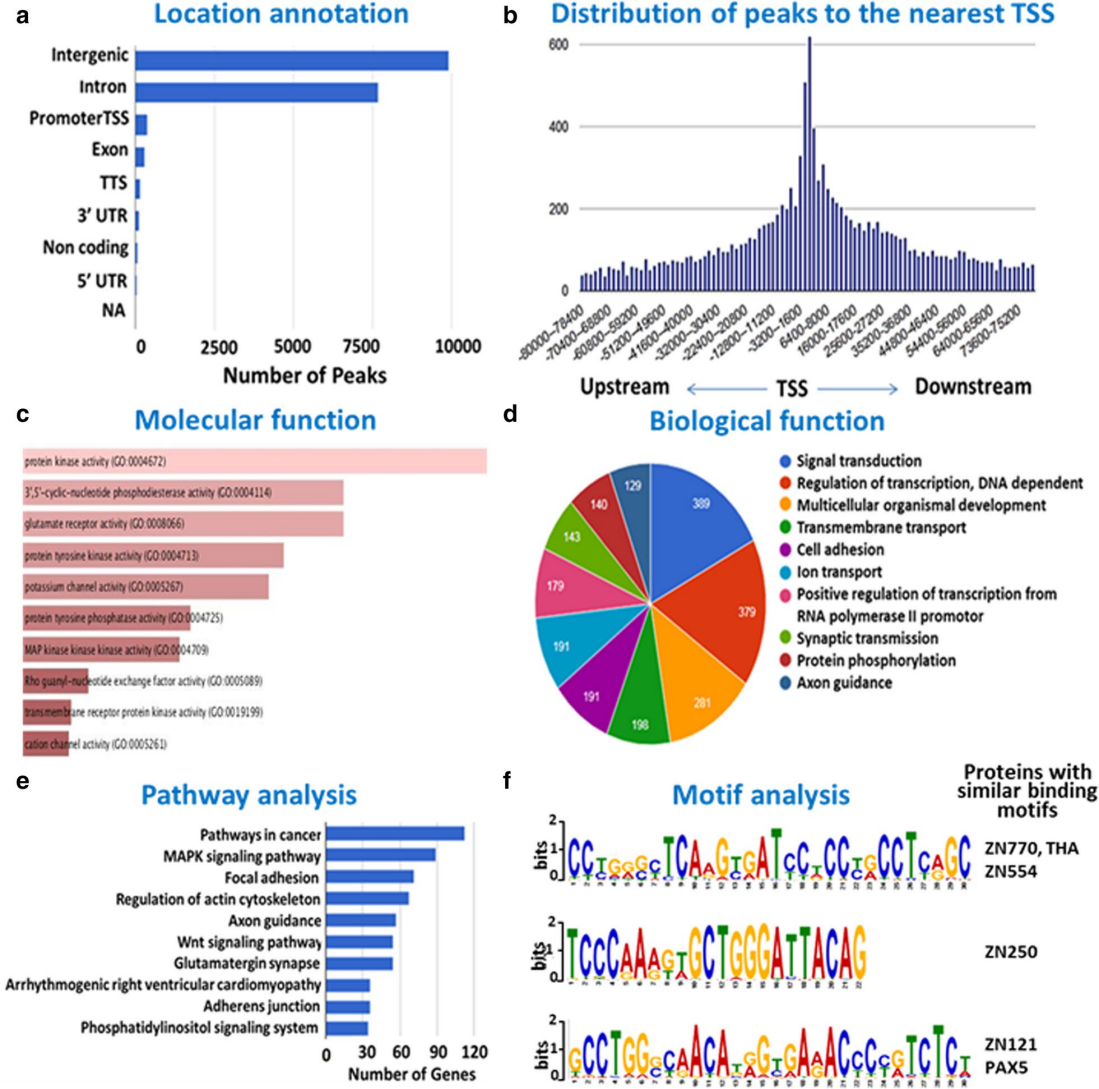
Bioinformatics and GO analysis: Peak location of TH2B enriched regions (**a**), Distribution of reads around Transcription Start site (**b**), Enriched biological and molecular function of TH2B associated genes (**c**, **d**), Most enriched pathway (**e**), and Three most enriched DNA motifs with which TH2B is associated (**f**)

The exhaustive list of TH2B associated genes is provided as Additional file 2. TH2B localization analyzed using ChIPseek [22] tool indicated that TH2B is present mainly in the intergenic and intronic regions, with modest enrichment at promoter, exon and TTS (Fig. 3a). Interestingly, significant number of peaks (representing TH2B enriched regions) were found to be distributed around TSS of genes (Fig. 3b). Regions observed to be enriched upstream and downstream relative to TSS are  presented as Fig. S1 in Additional file 3.

GO analysis using Genecodis 3 and EnrichR [23, 24] was performed for the genes associated with TH2B. Signal transduction (p* = 7.02E-65), regulation of transcription (p* = 4.42E-26) and multicellular organism development (p* = 3.97E-36) were found to be the most enriched biological function and Kinase activity was observed as a major molecular function (Fig. 3c, d). p* indicates corrected hypergeometric *p* value. The GO analysis raw data is provided as Additional files 4 and 5.

Pathway analysis featured pathways in cancer, MAPK signaling pathway, focal adhesion, and regulation of actin cytoskeleton amongst the most enriched pathway (Fig. 3e). Motif analysis using MEME ChIP [25] revealed several conserved DNA motifs to be associated with TH2B. The most enriched motifs observed to be associated with TH2B were similar to those reported to be associated with zinc finger proteins like ZNF770, ZNF554 and ZNF250 which are implicated in regulation of transcription (Fig. 3f). However, their role in testis remains unexplored as yet.

### TH2B enrichment at developmentally important loci and at genes involved in sperm function

**Fig. 4:**
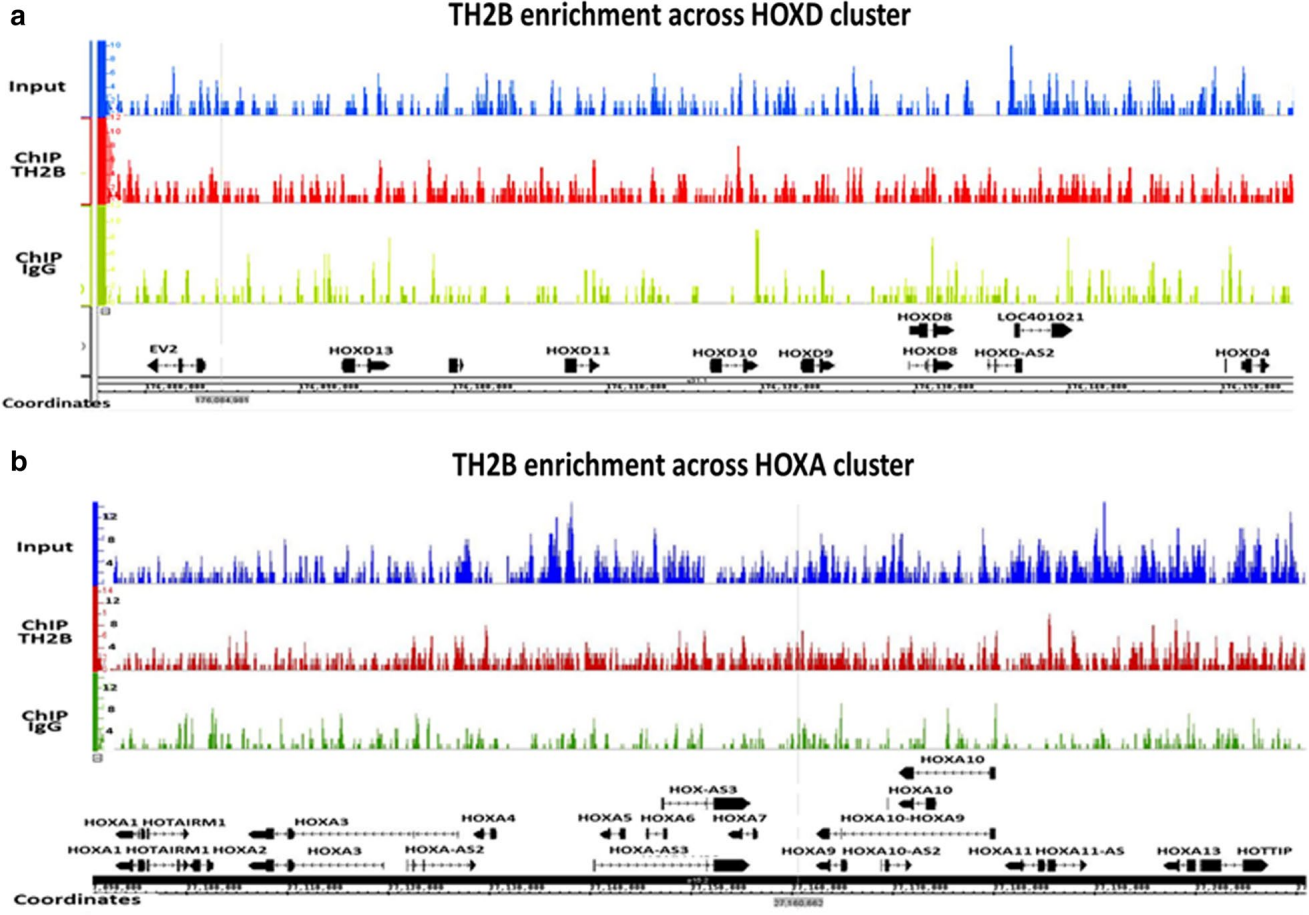
Integrated Genome Browser (IGB) view of HOXD and HOXA clusters showing enrichment of TH2B across both the clusters. The Y axis indicates number of reads while X axis denotes the chromosome coordinates

Enrichment of TH2B was observed at developmentally important loci like HOXA and HOXD (Fig. 4).

**Fig. 5:**
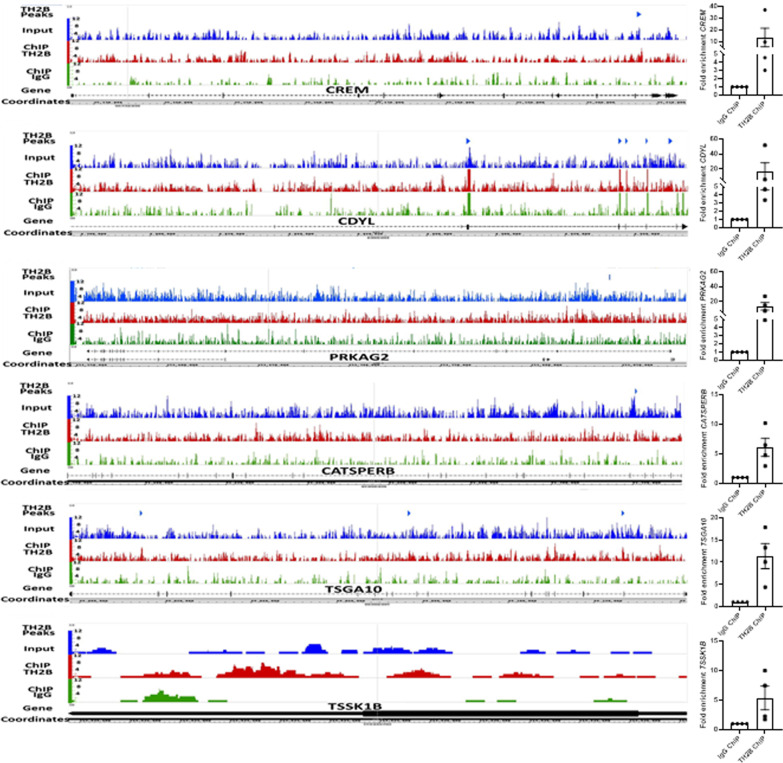
TH2B enrichment at genes involved in sperm function: IGB view of *CREM*, *PRKAG2*, *CDYL*, *TSGA10*, *TSSK1B* and *CATSPER B* identified to be associated with TH2B (left panel) and dot plot indicating fold enrichment of TH2B on the respective gene (right panel). The raw Cq values for genes associated with TH2B- or IgG- immunoprecipitated DNA were normalized to that of Input mononucleosomal DNA and then enrichment of the gene in TH2B immunoprecipitation over that in IgG immunoprecipitation, was calculated considering enrichment in IgG as one

TH2B was found to be enriched on 5527 genes, out of which 105 (1.9%) and 144 (2.6%) genes showed a direct involvement in sperm function, and early embryogenesis, respectively. We shortlisted few of the genes from these, on the basis of their importance in sperm function (Table 2). These were subsequently validated by ChIP-qPCR towards which primers were designed for the peak sequences associated with each gene (Table 4). The primers were designed such that the PCR product was less than 147 bp. TH2B-ChIP with sperm from a separate set of four fertile individuals was carried out and the immunoprecipitated DNA was used as template for PCR amplification of each gene (Fig. 5).

**Table 2:** Fold enrichment, biological processes and molecular functions of a few TH2B associated genes

Gene name	Protein name	Fold enrichment	p value	GO biological process	GO molecular function
*CREM*	cAMP-responsive element modulator	6.74	6 × 10^–4^	DNA-binding transcription factor activity, Regulation of transcription, Signal transduction,Multicellular organism development, Spermatogenesis	DNA binding, RNA polymerase II transcription factor activity, Transcriptional repressor activity, RNA polymerase II transcription regulatory region sequence-specific DNA binding
*PRKAG2*	5′-AMP-activated protein kinase subunit gamma-2 (AMPK gamma2)	5.9	7 × 10^–4^	ATP biosynthetic process, Fatty acid biosynthetic process, Intracellular signal transduction, Positive regulation of protein kinase activity, Regulation of glycolytic process, regulation of signal transduction	AMP-activated protein kinase activity, AMP binding, ATP binding, cAMP-dependent protein kinase regulator activity, Phosphorylase kinase regulator activity [GO:0008607]; protein kinase activator activity
*CDYL*	Chromodomain Y-like protein (CDY-like) (Crotonyl-CoA hydratase)	29.5	1 × 10^–5^	Negative regulation of peptidyl-lysine crotonylation, Random inactivation of X chromosome, Regulation of transcription, Spermatid development, spermatogenesis	Chromatin binding, Crotonyl-CoA hydratase activity, Methylated histone binding, Protein binding, Transcription corepressor activity
*CATSPERB*	Cation channel sperm-associated protein subunit beta (CatSper-beta)	5.06	7 × 10^–4^	Multicellular organism development, Response to progesterone, Sperm capacitation, Sperm-egg recognition	CatSpercomplex,Integral component of sperm membrane
*TSGA10*	Testis-specific gene 10 protein (Testis development protein NYD-SP7)	5.9	3 × 10^–4^	Cell projection assembly, Spermatogenesis	Protein binding
*TSSK1B*	Testis-specific serine/threonine-protein kinase 1	5.9	3 × 10^–4^	Intracellular signal transduction, Multicellular organism development, Protein phosphorylation, Spermatid development	ATP binding, Magnesium ion binding, Protein serine/threonine kinase activity

### Chromatin compaction in infertile individuals

The sperm chromatin compaction status of each fertile and infertile individual enrolled in this study was assessed by Acidic aniline blue dye staining (Histone status) and Chromomycin A3 detection (Protamine status). Semen characteristics of fertile and infertile individuals are summarized in Table 3 and the individual semen characteristics are detailed as Additional file 6.

**Table 3:** Semen characteristics and chromatin compaction status of fertile and infertile men

Group	% Progressive Motility	Concentration (× 10^6^)/ml	% Viability	% Aniline blue positive sperm	% CMA3 positive sperm
Fertile* (n = 14)	64 ± 10.4	43 ± 24.3	80 ± 7.5	17 ± 11.3	23 ± 11
Infertile					
Asthenozoospermia (n = 10)	14 ± 6.9^a^	18 ± 7.4^ab^	69 ± 8.7^a^	69 ± 11.2^a^	70 ± 16.3^a^
Oligozoospermia (n = 10)	43 ± 8.2^ab^	6 ± 2.7^a^	75 ± 8.0^b^	58 ± 16.2^a^	67 ± 19.3^a^
Oligoasthenozoospermia (n = 10)	17 ± 7.5^a^	4 ± 2.0^a^	60 ± 11.7^a^	69 ± 16.6^a^	77 ± 16.6^a^

*Fertile group (n = 14) encompasses men enrolled for the experiments involving ChIP-qPCR (n = 4) and qRT-PCR (n = 10)

^a^Significantly different as compared to fertile group

^b^Significantly different as compared to oligoasthenozoospermia group

**Fig. 6:**
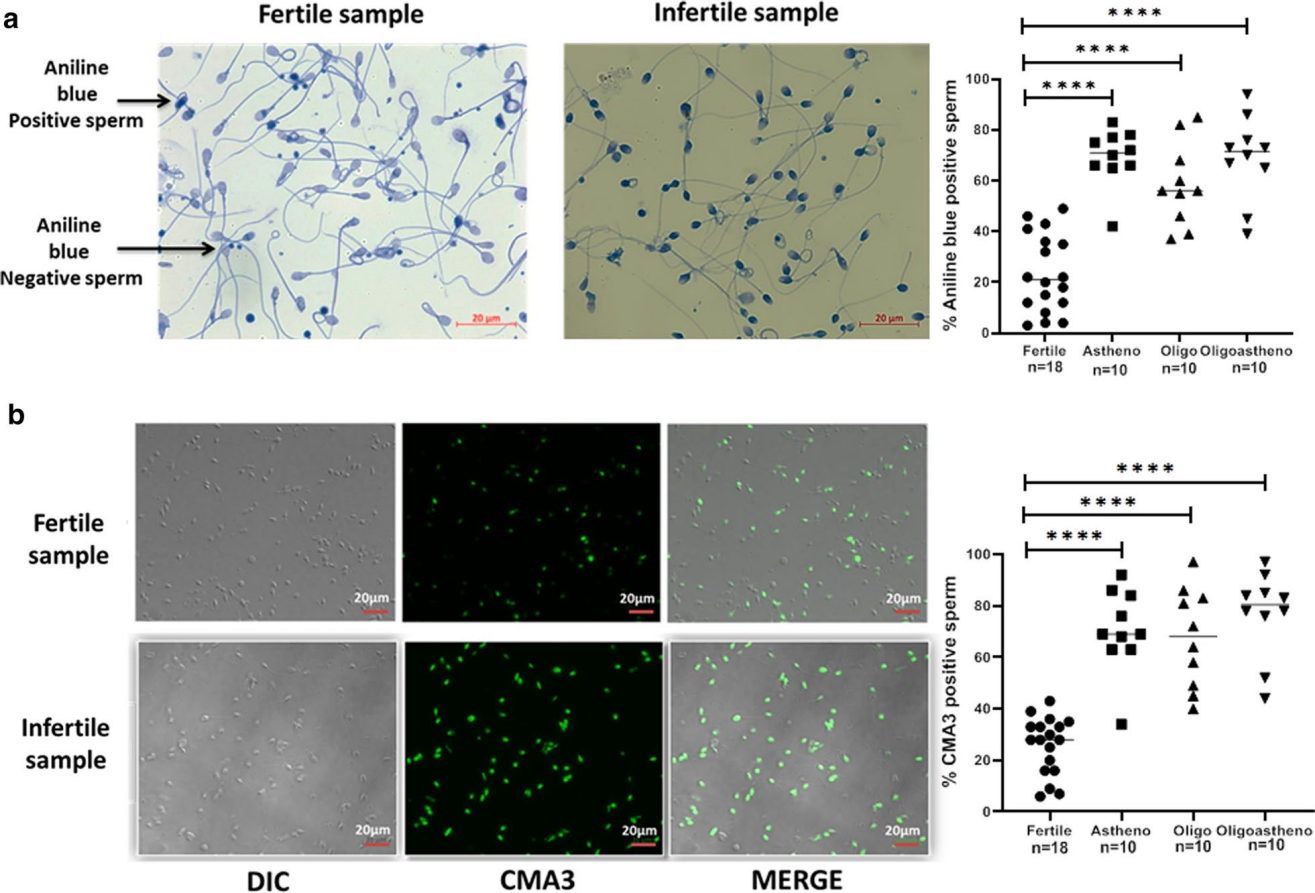
Chromatin condensation in sperm of fertile and infertile men: Histone status of sperm was assessed by Aniline blue staining. Representative picture showing darkly stained immature sperm nuclei and light stained mature nuclei in fertile and infertile semen sample (**a**-left panel). Protamine status was assessed by Chromomycin A3 staining. Representative picture of sperm from fertile and infertile semen samples shows bright fluorescing sperm nuclei which are CMA3 positive and dull fluorescing which are CMA3 negative (**b**-left panel). Dot plot shows percentage of aniline blue (**a**-right panel) and CMA3 positive sperm (**b**-right panel) in the fertile (n = 18) and infertile individuals, namely, men with Asthenozoospermia (n = 10), Oligoasthenozoospermia (n = 10) and Oligozoospermia (n = 10); p**** < 0.0001

Aniline blue binds lysine rich histones preferentially over arginine rich protamines. Darkly stained sperm nuclei indicate higher histone retention and thus chromatin immaturity while lightly stained sperm nuclei indicates normal histone retention and thus chromatin maturity. Percentage of aniline blue positive sperm was significantly higher (p*** < 0.0001) in infertile men with Asthenozoospermia, Oligozoospermia and Oligoasthenozoospermia, as compared to that in the fertile individuals (Fig. 6a).

CMA3 competes with protamine to bind to the minor groove of DNA, hence more binding of CMA3 represents lower protamine levels and vice versa. CMA3 positive, bright fluorescing, sperm nuclei represent immature chromatin condensation while CMA3 negative represent mature chromatin state. Percent CMA3 positive sperm were significantly higher in all subcategories of infertile individuals as compared to that in the fertile individuals (Fig. 6b).

### Expression profile of TH2B associated genes in sperm of fertile and infertile men

**Fig. 7:**
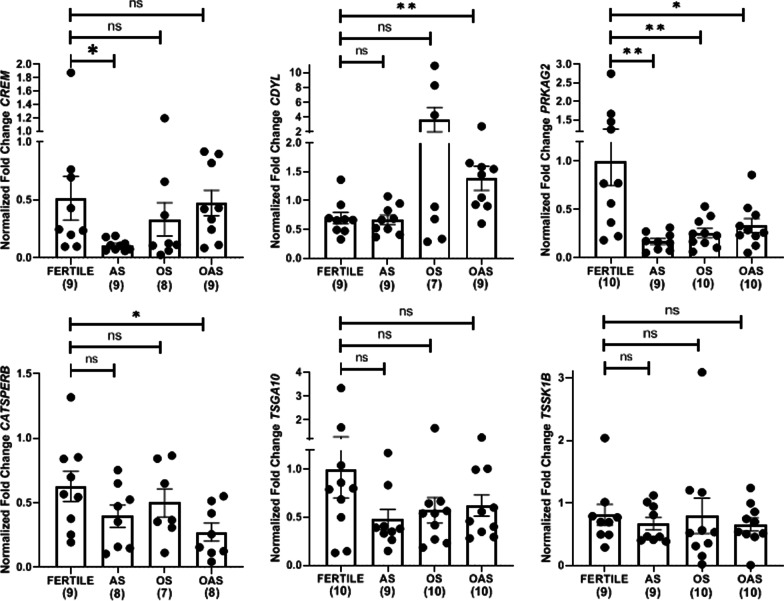
Expression profile of TH2B associated genes in sperm of fertile and infertile men. Normalized fold change in transcript of *CREM*, *CDYL*, *PRKAG2*, *CATSPER B*, *TSGA10*, and *TSSK1B* in fertile and infertile groups (AS- Asthenozoospermia, OS- Oligozoospermia and OAS- Oligoasthenozoospermia). p* and p** indicate *p* < 0.05, and *p* < 0.01, respectively; ‘ns’ denotes no significant difference. Data are plotted as mean ± SEM. Dot represents fold enrichment of a transcript in an individual. The numbers in parenthesis indicates number of samples included in the analysis for that particular group for a given gene

The relative abundance in transcripts of few TH2B associated genes- *CREM, CDYL, PRKAG2, CATSPERB, TSGA10* and *TSSK1B* was studied in sperm of fertile and infertile men with asthenozoospermia-, oligozoospermia- or oligoasthenozoospermia. The results indicated subgroup specific alteration in transcripts of some of the genes. Relative expression of *CREM* was found to be significantly lower in men with asthenozoospermia (*p* = 0.047) while it was comparable in men with oligozoospermia (*p* = 0.46) and oligoasthenozoospermia (*p* = 0.85) to that of fertile men. *CDYL* expression was found to be significantly higher in men with oligoasthenozoospemia (*p* = 0.009), while it was not altered in the other two subgroups of infertile men. *PRKAG2* expression was significantly reduced in men with asthenozoospermia (*p* = 0.007), oligozoospermia (*p* = 0.009), and oligoasthenozoospermia (*p* = 0.02). *CATSPERB* expression was observed to be reduced in men with oligoasthenozoospermia (*p* = 0.02). *CATSPERB* expression was apparently reduced in sperm of men with asthenozoospermia, but it was not statistically significant (*p* = 0.14) while in men with oligozoospermia it was unaltered. Relative expressions of *TSGA10* and *TSSK1B* did not differ in any of the infertile subgroups as compared to that in the fertile group (Fig. 7).

### Correlation analysis

**Fig. 8:**
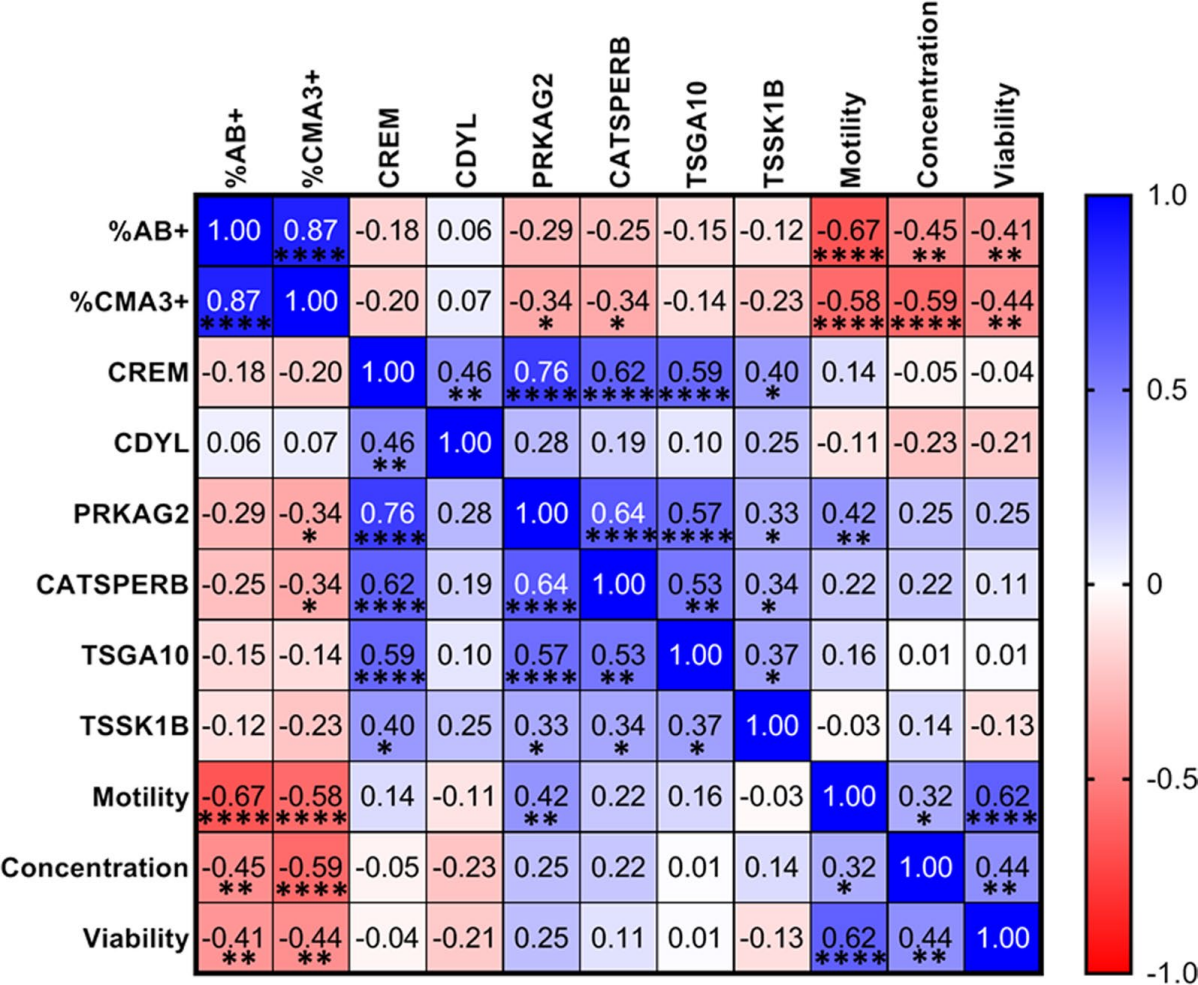
Correlation analysis between sperm chromatin compaction, various semen parameters and transcript levels of TH2B associated genes. Heatmap showing Spearman correlation analysis between percent aniline blue positive- (%AB+), percent CMA3 positive (%CMA3+), sperm motility, -concentration,-viability and transcript levels of *CREM*, *CDYL*, *PRKAG2*, *CATSPERB*, *TSGA10* and *TSSK1B*. p* < 0.05, p** < 0.01, p*** < 0.001 and p**** < 0.0001

Spearman correlation analysis was carried out between percent aniline blue positive sperm (%AB+), percent CMA3 positive sperm (%CMA3+), sperm motility-, concentration-, viability and transcript levels of *CREM, CDYL, PRKAG2, CATSPERB, TSGA10,*and *TSSK1B*. A significant negative correlation was observed for percentages of AB+ and CMA3+ sperm with sperm motility and concentration. Transcript levels of *PRKAG2* and *CATSPERB* negatively correlated with %CMA3+ sperm while for *CREM, CDYL TSGA10,* and *TSSK1B* no significant correlation was noted. Transcript levels of *CREM* positively correlated with transcript levels of *CDYL, PRKAG2, CATSPERB, TSGA10,* and *TSSK1B*. PRKAG2 expression positively correlated with sperm motility (Fig. 8)

## Discussion

The genome-wide localization of TH2B in primary spermatocyte (of mouse), was recently published [26]. However the biological relevance of the presence of this major histone variant in sperm is not fully understood. From studies of TH2A/TH2B double knockout mice, we know that male mice are sterile and chromatin compaction is drastically altered, thus implying the importance of these testis specific histone variants in male fertility [20]. Studies by Padavattan et al. [19] indicate that presence of TH2B and TH2A generates more dynamic nucleosomes, leading to an open chromatin structure, which is characteristic of a transcriptionally active genome. Hence, the presence of TH2B in mature sperm is intriguing given that mature human sperm are transcriptionally and translationally silent. To address its role in sperm, we investigated the genome-wide localization of TH2B in sperm of fertile men. This is important to know as most of the canonical H2B is replaced by TH2B during spermiogenesis [27].

Histone variants are non-canonical variants of histones with only few amino acid differences from their canonical counterparts. TH2B differs from somatic H2B at its N-terminal (S2A in Additional file 3). Using chromatin immunoprecipitation followed by high- throughput sequencing approach, we identified the genomic loci associated with TH2B in fertile human sperm. TH2B was found to be mostly in intergenic and intronic regions (Fig. 3a, b). Similar observations were made by Yamaguchi et al. for Histone H4 wherein H4 localization was observed majorly to intergenic regions [28]. A landmark study by Hammoud et al., in 2009 showed for the first time by MNase-seq, the enrichment of retained nucleosomes at developmentally important loci, suggesting their probable role post fertilization. When the same approach was adopted by different investigators, contradictory observations were noted. These may be due to variation in two critical steps 1) Micrococcal nuclease digestion and 2) Bioinformatics pipeline used for data analysis. As sperm DNA compaction is unique and complex, we first optimized conditions to get a precise mononucleosomal pool while avoiding digestion of protamine bound DNA. Immunoprecipitation of a specific histone variant can be challenging due to high similarity with its canonical counterpart. In our study, establishment of the antibody specificity followed by ChIP-Western validation confirmed the specific immunoprecipitation of TH2B (S2B in Additional file 3).

The retained histones are generally observed with the genes or gene clusters involved in early embryogenesis. Data from ChIP on chip analysis with TH2B suggested promoters of genes involved in sperm function to be associated with TH2B. However this interesting observation was not explored further [4]. We observed TH2B retention on developmentally important loci like HOXA and HOXD (Fig. 4) as well as on genes involved in sperm function. The entire data with all the genes and their respective ontologies is provided as Additional file 2. This includes the TH2B associated genes involved in early embryo development as well as those involved in sperm function. In this paper, we discuss our observations on the genes involved in sperm function. We validated the TH2B enrichment on *CREM, CDYL, PRKAG2, CATSPERB, TSGA10* and *TSSK1B* by ChIP-qPCR (Fig. 5).

Histones are the key epigenetic players and regulate chromatin function. Histone variants and modifications occurring on them are found to be associated with specific biological processes like DNA strand repair, meiotic sex chromosome inactivation, and transcription. TH2A and TH2B are implicated in active transcription, and H3K4me3 and H3K27me3 are found to be associated with active and repressed state of the genome, respectively. Even loss of function of chromatin remodeling factors or enzymes carrying out particular histone modification can lead to altered transcriptome in sperm [29–33]. This implies a relationship between chromatin state during spermiogenesis and timely transcription activity.

To investigate this possibility, we studied the relative expression of few TH2B associated genes in infertile men with abnormal chromatin packaging. We hypothesized that alteration in either incorporation or eviction of TH2B during the course of spermiogenesis may lead to abnormal chromatin packaging and transcription. This hypothesis was based on the observations by Sendler et al. [34], wherein a strong correlation was noted between H3K4me3 bearing genes and their cognate transcripts in mature sperm. This suggests that these genes, at the time of spermiogenesis, may be bearing the histone activation mark which was retained in mature sperm.

Subgroup specific altered expression in infertile men was observed for few TH2B associated genes (Fig. 7). Transcripts for CREM, which is the major transcription factor involved in the expression of a number of genes during the post meiotic stage of sperm development, was found to be significantly low in sperm of men with asthenozoospermia, suggesting that proteins required for sperm motility may be under the control of CREM or its associated transcriptional network. Correlation analysis of our data revealed a positive correlation of transcript levels of *CREM* with transcript levels of *CDYL, PRKAG2, CATSPERB, TSGA10,* and *TSSK1B* suggesting regulation of these genes by CREM (Fig. 8). This is further substantiated by the transcriptome data from *Crem* deficient mice, which shows altered expression of *CDYL*, *CATSPER1* and *TSSK1* [35]. Though CREM occupies more than 9000 loci in the developing spermatid, only a subset of genes were altered in *Crem* deficient mice, which suggests that alternative factors may be controlling expression of these unaltered genes [36]. Apart from CREM, several other proteins are now reported in the regulation of transcription in sperm [1].

Studies with human CDY and mouse CDYL proteins demonstrate their histone acetyltransferase activity, especially on histone H4. The expression and localization of CDYL coincides with H4 hyperacetylation during spermatogenesis which suggested that Cdyl may be involved in histone to protamine transition via H4 hyperacetylation [37]. This was also supported by the observation that lack of *Cdyl* leads to dysregulated histone replacement in the testis of *Cdyl* transgenic mice. A recent study demonstrated that CDYL regulates the expression of sex chromosome-linked escaped genes in postmeiotic spermatogenic cells by acting as a crotonyl-CoA hydratase [30]. In a cell-based model of transcriptional activation, increasing or decreasing the cellular concentration of crotonyl-CoA led to enhanced or diminished gene expression, respectively, and these crotonylated histones were present in regulatory region of active genes, which again suggest involvement of histone crotonylation in active transcription [38]. We have previously reported Cdyl to be potential tubulin acetyltransferase in rat sperm [39]. Recently, we have reported HDAC6, the tubulin specific deacetylase to be reduced in sperm of men with asthenozoospermia [40]. In light of this information, increased expression of *CDYL* observed in all the subgroups of infertile men with significantly increased expression in men with oligoasthenozoospermia group where both motility and sperm numbers are affected, is noteworthy (Fig. 7). The synergistic effect of histone deacetylase and histone acetyl transferase may influence microtubule dynamics and consequently sperm function.

AMP activated protein kinase (AMPK) has been reported to be essential for sperm motility in boar spermatozoa [41]. AMPKα1 knockout mice sperm showed asthenozoospermia characteristics and structural abnormalities, however their chromatin compaction status was not reported [42]. Under stress conditions, AMPK is known to directly phosphorylate H2B in cells and facilitate expression of stress related proteins implying AMPK to be a phosphorylating kinase for H2B [43]. We observed Protein Kinase AMP-Activated Non-Catalytic Subunit Gamma 2 (PRKAG2) to be reduced in sperm of men with asthenozoospermia (Fig. 7). Correlation analysis revealed a positive correlation of *PRKAG2* with sperm motility (Fig. 8). In metabolic stress condition, gamma subunit of AMPK binds to AMP and regulates activity of catalytic alpha subunit to bring out the phosphorylation of its substrate [44]. In light of this knowledge and our previous observation of reduced pTH2B in men with poor sperm motility [45], we believe that AMPK may be the putative kinase for TH2B phosphorylation in sperm and there may likely be a link between TH2B phosphorylation and sperm motility. Functional implications of this altered phosphoswitch need to be explored.

CatSper channel plays a pivotal role in attaining hyperactivated motility during sperm capacitation [46]. CatSper channel is made up of 10 subunits of which six are pore forming catSper alpha while catSperbeta is one of the remaining auxiliary subunits. CatSperb is absent in sperm from mice lacking CatSper1, suggesting that the expression of CatSperb and CatSper1 is linked [47]. CatSper knockout males show poor sperm motility and are sterile [48]. At transcript level we observed *Catsperb* to be reduced in all the subgroups of infertile men, with significant reduction seen in men with oligoasthenozoospermia.

**Fig. 9:**
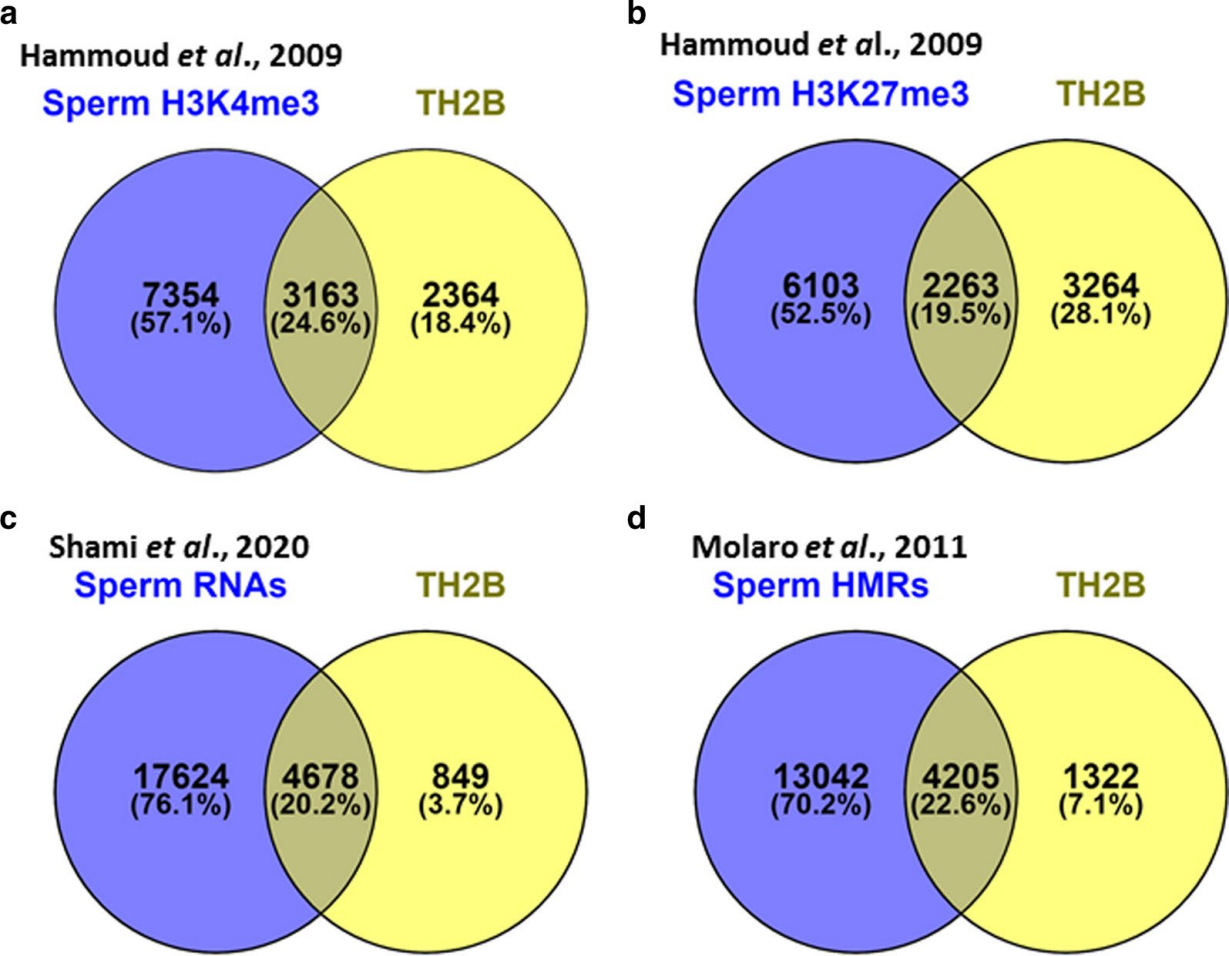
Comparative analysis of TH2B associated genes for histone methylation marks, transcripts and hypomethylated regions (HMRs) in human sperm. Number of TH2B associated genes bearing activating mark (**a**) and repressing mark (**b**), the presence of their cognate transcript (**c**) and HMRs (**d**) in human sperm were determined by analyzing our TH2B ChIP dataset against datasets from H3K4me3- and H3K27me3 ChIP-seq data sets of Hammoud et al. [4], sperm RNA data set of Shami et al. [51], and sperm HMR data set of Molaro et al. [52]

Distinct set of genes in sperm chromatin retain specific histone marks which decide fate of that gene. The genes involved in sperm function bear the activation histone mark H3K4me3 and those required during early embryogenesis retain repressing histone mark H3K27me3. Most of the genes retaining nucleosomal structure contain bivalent promoters which signify presence of both activation and repressing mark at the same promoter. Such types of promoters are frequently found in embryonic stem cells. Classically, it was thought that bivalency imposes repressive state on gene which gets activated in the presence of a signal when the gene expression is needed. However, very recently, it was suggested that such promoters may protect the gene from irreversible silencing through inhibition of hypermethylation of the gene [49]. The role of TH2B in mature sperm is not clear but can be speculated by investigating its coexistence with histone activating and/or repressing marks. Towards this, we retrieved the ChIP-seq BED files of H3K4me3 and H3K27me3 in mature sperm from Hammoud et al., 2009. The genome coordinates were uplifted to hg19 using UCSC genome browser and then gene annotation was carried out. Venny [50] was used to know the gene overlap between the datasets studied. Most of the TH2B associated genes appeared to have bivalent histone modifications (Fig. 9a, b). Out of the six genes which were investigated in this study, four genes namely *CREM, CDYL, PRKAG2 and CATSPERB* are found to be associated with both histone activation (H3K4me3) and repression marks (H3K27me3) in human sperm. TSGA10 bears H3K27me3 mark while TSSK1B has not been reported to be associated with any mark. Interestingly, a significant number of TH2B associated genes were identified to be hypomethylated and their RNA were also present in sperm (Fig. 9c, d). Hypomethylation of a gene is generally associated with its transcriptionally active state. Mature sperm is regarded as transcriptionally and translationally inactive. It thus appears that the genes which retain nucleosomal structure in sperm may be epigenetically programmed during the course of spermatogenesis and these marks are retained in mature sperm.

Spearman correlation analysis revealed a positive correlation between chromatin compaction and sperm motility, as well as concentration as also evident from Fig. 6 (Fig. 8).

Unlike rodent sperm, the human sperm population is highly heterogeneous. Consequently, high standard deviations due to inter sample variation with respect to sperm chromatin compaction status were observed. This is an inherent limitation with all studies employing human sperm. Additionally, enrichment of mononucleosomal DNA from individual samples does not provide sufficient DNA for high-throughput sequencing hence the mononucleosomal DNA from spermatozoa of four fertile men had to be pooled.

Abnormal chromatin packaging during spermiogenesis may reflect as altered transcriptome in sperm. Given that sperm are transcriptionally and translationally inactive, the sperm transcriptome can provide an insight about the transcriptional events occurred during spermatogenesis. Recent evidences also suggest that sperm RNAs get transferred to the oocyte during fertilization and influence early embryo development [53]. Hence a qRT-PCR approach was employed by us to quantify the transcript abundance, as it is the most widely used technique to study relative abundance of sperm transcripts [54–57]. All precautions were taken during real time PCR quantification. Firstly, oligo-dT along with random primers in 3:1 proportion were used during cDNA synthesis, thus ensuring that any transcript which is not full length still get detected during real time amplification. Further to eliminate the chances of non-specific amplification and real time sensitivity issues, TaqMan chemistry was employed for transcript quantification. It is possible that sperm RNA may be degraded depending on the duration of their storage in the epididymis and this could influence detection of transcripts on qRT-PCR. To minimize this possibility, in the present study, it has been ensured that all semen samples were collected following a strict abstinence period of 3–4 days, as per WHO criteria. In this context, there is a recent evidence of the presence of full length transcripts in sperm [58].

Whilst the present study explored the TH2B associated genes involved in sperm function, it will be interesting to investigate significance of TH2B associated genes in the development of the early embryo. The effect of abnormal sperm chromatin packaging on transcription of these genes during early embryo development may reveal the importance of nucleosome retention on these genes in sperm. Studies in this direction have been initiated.

In conclusion, we studied the genome-wide distribution of TH2B in fertile sperm and found that TH2B was present on loci important for early embryogenesis and sperm function. TH2B was associated with ~ 5% of the total genome. All chromosomes except sex chromosomes showed varied distribution of TH2B. TH2B was found to be more enriched on genes of chromosome 4, 18, 3 and 2. Positive correlation between AMPK and sperm motility emphasizes a possible link between phosphorylated TH2B and sperm motility. Sperm chromatin compaction positively correlated with sperm motility, and concentration. Infertile individuals having defective sperm chromatin compaction showed altered expression of TH2B associated genes indicating involvement of TH2B in transcriptional regulation of these genes in post meiotic male germ cells.

## Material and methods

### Processing of Human semen samples

Human spermatozoa from fertile and infertile individuals were used for this study. The use of human semen samples was approved by ICMR- National Institute for Research in Reproductive Health Clinical Ethics Committee, Mumbai, India (Project No. 305/2017). Semen samples from fertile and infertile individuals (men with Asthenozoospermia, Oligozoospermia and Oligoasthenozoospermia) were collected following 3–4 days of abstinence. In the fertile group, men who had fathered a child in the preceding one year and had normal semen parameters were included. In the infertile group, men who suffered from primary infertility or secondary infertility and were having asthenozoospermia, oligozoospermia (but count not less than 1 × 10^6^ /ml) or oligoasthenozoospermia were included. Semen samples showing hyper viscosity, or infection as indicated by the presence of pus cells, were not included in the study. Semen analysis was carried out as per the WHO 2010 guidelines for semen analysis [59]. Briefly, Semen samples were allowed to liquefy for 30–45 min at RT following which spermatozoa motility, count and viability were noted. A small fraction of each sample was used to study the chromatin condensation status by Aniline blue and CMA3 staining. For experiments involving ChIP-seq and validation of high-throughput data, Puresperm density gradient purification of spermatozoa was done for the semen samples of fertile men. Briefly, 2-3 ml of semen sample was diluted in HTF medium (Merk-millipore, Darmstadt, Germany) containing 5% human albumin (Sigma-aldrich, St. Louis, MO, USA: HTF-albuman medium), overlaid on 80% Puresperm density gradient (Nidacon, Molndal, Sweden) prepared using HTF- albuman medium and centrifuged at 350 g for 20 min. The pelleted fraction containing motile spermatozoa was washed thrice with 0.1 M PBS to eliminate any traces of puresperm solution.

Semen samples used to study gene transcript abundance in sperm of fertile- and infertile men, were not subjected to puresperm gradient.

### Aniline blue staining

Sperm histones were stained with Acidic aniline blue stain (Himedia, Mumbai, Maharashtra, India) as described by Sellami et al. [60]. Briefly, sperm smears were fixed with 3% Glutaraldehyde for 30 min, stained with 5% Acidic Aniline blue solution for 5 min at RT and observed under an oil immersion objective on Axio Observer Carl Zeiss microscope system (Carl zeiss, Oberkochen, Germany). 200 spermatozoa were counted and categorized as darkly stained (Aniline blue positive) and lightly stained (Aniline blue negative).

### CMA3 Staining

The Chromomycin A3 (CMA3) staining for protamine status was carried out using a published protocol [61] with modifications. Briefly, the sperm smears were fixed using Carnoy’s solution (Methanol: Acetic acid, in 3:1 v/v ratio) for 30 min at 4 °C. Slides were incubated with Acid detergent solution, pH 1.2 containing 0.08 N HCl, 0.15 M NaCl, 0.5% Triton X100 for 30 min at RT followed by staining with 0.25 mg/ml CMA3 (Enzo, Farmingdale, NY, USA) prepared in 0.1 M Citric acid, pH 7 containing 0.2 M Na_2_HPO_4_ and 0.025 M MgCl_2_ (McIlvaine buffer) for 30 min at RT in dark conditions. The slides were washed using McIlvaine buffer, mounted with Prolong gold antifade reagent (Life technologies, Carlsbad, California, USA) and observed at 630X magnification at excitation wavelength of 488 nm. 200 spermatozoa were counted; the brightly fluorescing sperm were categorized as CMA3 positive and dimly fluorescing as CMA3 negative.

### Isolation of mononucleosomes

Chromatin preparation of sperm DNA to yield pure mononucleosomal fraction was carried out as per the protocol described by Hisano et al. [62]. Briefly, Puresperm purified spermatozoa were resuspended in 15 mM Tris–HCl buffer (pH 7.5) containing 60 mM KCl, 5 mM MgCl_2_, 0.1 mM EGTA, 0.3 M sucrose and 10 mM DTT. Cells were lysed for 30 min. using NP-40 and Sodium deoxycholate (DOC) at a final concentration of 0.5% (vol/vol) and 1% (wt/vol), respectively. Sperm chromatin was subjected to 5, 15, or 30 Units of micrococcal nuclease (MNase; New England Biolab, MA, USA) per 2 million sperm, at 37 °C for 5 min. Reactions were terminated using 0.5 M EDTA, DNA was isolated using Exgene™ cell SV mini kit (GeneAll, Dongnam, Songpa, Seoul, Korea), electrophoresed on 12% PAGE, stained with EtBr and visualized using Gel documentation system (Syngene, Cambridge, UK). The reaction containing 5 U MNase showed prominent mononucleosomal band at around 147 bp as compared to digestions using 15 or 30 U MNase (S2C of Additional file 3). The lower intensity of mononucleosomal DNA band seen with 15 Units of MNase may represent over digestion of mononucleosomal DNA while presence of partially digested higher molecular weight DNA seen with 30 Units may indicate digestion of protamine bound DNA. Thus for the subsequent experiments, 5 Units of MNase was used per 2 million sperm to isolate mononucleosomes.

### Chromatin immunoprecipitation of TH2B and processing of high-throughput data

A pure preparation of human spermatozoa was obtained to avoid contamination of any other cell type by Puresperm density gradient centrifugation. Chromatin immunoprecipitation of TH2B from sperm of four fertile individuals was carried out as per the protocol described by Hisano et al. [62] with minor modifications. Briefly, sperm DNA was digested using 5 Units of MNase per 2 million sperm. Mononucleosomes from 12 million cells were used for immunoprecipitation using 4 μg of Anti- TH2B antibody (Merck-millipore, Darmstadt, Germany, 07–680). An equivalent amount of IgG (Merck-millipore, Darmstadt, Germany, 12–370) was used as Isotype control for immunoprecipitation. Mononucleosomal DNA isolated from 2 million sperm cells served as input. A small fraction of immunoprecipitation complex was subjected to protein elution in 2X Laemmli solution by heating at 95 °C for 10 min followed by western blot detection (ChIP- Western) of TH2B and immunoprecipitated DNA was isolated from the rest. DNA was electrophoresed on 5% PAGE and stained with ethidium bromide. The band at ~ 147 bp corresponding to mononucleosomal DNA was excised and DNA was precipitated. 5 ng of DNA each from Input, TH2B-ChIP and IgG-ChIP was used for library preparation. High-throughput paired end sequencing was carried out on Illumina NextSeq500 platform at Sandor Lifesciences Pvt. Ltd. Hyderabad, India. Processing and quality control of raw reads was performed using NGSqctoolkit. Bowtie-0.12.9 was used for ungapped alignment of processed reads to the reference genome hg19. The resulting SAM files (Alignment files) and their binary version (BAM files) were processed by Samtools-0.2.7a and BED Tools Version 2.17.0, respectively. MACS-1.4.2 was used for calling peaks and identification of enriched regions. Finally, PeakAnalyzer-1.4 was used for annotation of peaks.

### ChIP-qPCR

The peaks sequences corresponding to a few genes were retrieved from the BED file. These sequences were used to design gene specific primers using Primer 3 tool. The primers were designed such that the corresponding amplicon should be less than 147 bp, which is the size of mononucleosomal DNA. The sequences of the primers used and annealing temperature for each gene is specified in Table 4.

**Table 4:** Sequences of the primers used and annealing temperature for each gene

Gene	Forward primer	Reverse primer	Annealing temp. (°C)
*CREM*	CCACATTACTGAATATGGGTGCT	TTTCTAAGTGCAGAAACATGCCT	58
*CDYL*	GGACTTCAAAGTTGGGGGCA	GAGGGTCTGTCAGCTTGTGA	60
*PRKAG2*	TGAGCCTTCAGTGAGTGGTA	CCATCCTAACCTTCAGGAAGCA	60
*CATSPERB*	GCACCGTATGGTGTGGACTA	CCCTCCAGACAAAGCACCAT	60
*TSGA10*	GGTGTCTGCTAATTGCCAGGT	GATAGGAGGTTTGGGCCACG	60
*TSSK1B*	CCAACGGGATCTTGCTGAAC	AGCATATCCATACGCAGAACCAT	62

PCR amplifications of *CREM, CDYL, PRKAG2, CATSPER B, TSGA 10* and *TSSK1B* were carried out using 0.1pMol of their respective forward- and reverse primers. Initial denaturation of template was done at 95 °C for 5 min followed by 40 cycles of denaturation at 95 °C for 1 min, annealing at their respective annealing temperatures for 15 s and extension at 72 °C for 25 s. Final extension was carried out at 72 °C for 10 min. Melt curve analysis was carried out for specificity of the amplicon. Enrichment of the corresponding amplicon in TH2B-ChIP DNA over that in IgG-ChIP DNA was calculated [63]. Towards this, both the ChIP DNA fractions were first normalized to input DNA fraction by the formula ΔCq [normalized ChIP] = Cq [ChIP] − (Cq [Input] − Log_2_input dilution factor), where input dilution factor was 10 as 10% of IP reaction was used as input. The IgG enrichment was then considered to be one and fold enrichment in TH2B was calculated as

2^−[ΔCq normalized ChIP TH2B − ΔCq normalized ChIP IgG]^.

### SDS Polyacrylamide Gel Electrophoresis (SDS-PAGE) and Western blot analysis

Immunoprecipitation complexes obtained from ChIP-TH2B/ ChIP-IgG were eluted in 2X Laemmli buffer and electrophoresed on 15% SDS-PAGE at 100 V for 2.5 h. Proteins were transblotted on Nitrocellulose membrane (Pall bioscience, Pensacola, FL, USA) at 100 V for 1 h 15 min. Non-specific binding to the membrane was blocked with 5% NFDM incubated on a rocker for 1 h at RT. After blocking, the membrane was incubated with anti-TH2B antibody diluted 1:5000 in 1% NFDM and kept overnight at 4 °C with constant rocking. The following day, blots were washed thrice with 0.1% PBST for 5 min and incubated with 1:3000 diluted Protein-G HRP (Thermo fisher scientific, Waltham, MA, USA), which served as secondary antibody, for 45 min with constant rocking. The blots were washed thrice with 0.1%PBST for 5 min and developed using Western Blot Chemiluminescence HRP substrate (TAKARA BIO INC, Otsu, Shiga, Japan).

### qRT-PCR

Total RNA was extracted from sperm of 10 fertile and 30 infertile men (10 each of men with Asthenozoospermia-, Oligozoospermia- and Oligoasthenozoospermia) by TRIzol® (Invitrogen, Carlsbad, California, USA) following the protocol described by the manufacturer. 500 ng of RNA from each sample was treated with DNase1 (Thermo fisher scientific, Waltham, MA, USA) and used for cDNA conversion using first strand cDNA synthesis kit (TAKARA BIO INC, Otsu, Shiga, Japan). The expression of each gene in all samples was assayed by TaqMan chemistry using the TaqMan™ Universal Master Mix II with UNG and FAM-labeled probes (ThermoFisher Scientific, MA, USA). 18S rRNA was used as housekeeping control. The abundance of gene transcript in sperm of infertile men vis a vis sperm of fertile men was calculated using 2^−ΔΔCq^ method. In a few samples from the infertile group despite the 18S RNA being consistently detectable, the transcript level of a particular gene was below the detection limit. Such samples had to be excluded from the analysis for that gene.

## Supplementary Information

**Additional file 1.** Chromosome-wise distribution of TH2B associated genes.

**Additional file 2.** Gene ontology analysis of TH2B associated genes; TH2B associated genes involved in sperm function and embryo development. 

**Additional file 3.** Location of TH2B enriched regions from TSS (S1); Specificity of TH2B antibody and MNase digestion of sperm DNA (S2).

**Additional file 4.** GO- Biological process of TH2B associated genes.

**Additional file 5.** GO- Molecular function of TH2B associated genes.

**Additional file 6**. Semen parameters of fertile and infertile samples

## Data Availability

The processed data is provided as additional files with this article and raw datasets generated and/or analysed during the current study are available in the NCBI-SRA repository, https://www.ncbi.nlm.nih.gov/sra/PRJNA715579

## Abbreviations

H2B: Histone 2B
H4: Histone 4
IVF: In-vitro Fertilization
ICSI: Intracytoplasmic Sperm Injection
TH2B: Testis Specific Histone H2B
TH2A: Testis Specific Histone H2A
pTH2B: Phosphorylated Testis Specific Histone H2B
ChIP: Chromatin Immunoprecipitation
GO: Gene Ontology
PCR: Polymerase Chain Reaction
qPCR: Quantitative real time PCR
qRT-PCR: Quantitative Reverse transcriptase real time PCR
Hg19: Human Genome 19, Genome Reference Consortium Human Build 37 (GRCh37)
MNase: Micrococcal Nuclease
UTR: Untranslated Region
TSS: Transcription Start Site
TTS: Transcription Termination Site
HTF: Human Tubular Fluid
H3K4me3: Histone H3 tri-methylated at Lysine 4
H3K27me3: Histone H3 tri-methylated at Lysine 27
H3K9me3: Histone H3 tri-methylated at Lysine 9
UNG: Uracil-N- glycosylase
FAM: Fluorescein amidites
IgG: Immunoglobulin G
AB: Aniline Blue
CMA3: Chromomycin A3
miRNA: Micro RNA
MAPK: Mitogen-activated protein kinase (MAPK)
ZNF: Zink finger protein
AS: Asthenozoospermia
OS: Oligozoospermia
OAS: Oligoasthenozoospermia
HMR: Hypomethylated regions
HOXA: Homeobox A Cluster
HOXD: Homeobox D Cluster
CREM: CAMP Response Element Modulator
CDYL: Chromodomain Y-like
PRKAG2: 5′-AMP-activated protein kinase subunit gamma-2 (AMPK gamma2)
CATSPERB: Cation channel sperm-associated protein subunit beta (CatSper-beta)
TSGA10: Testis-specific gene 10
TSSK1B: Testis-specific serine/threonine-protein kinase 1
NP40: Nonylphenoxypolyethoxylethanol
DOC: Sodium salt of Deoxycholic acid
