# Supplementary material for: Epigenetic landscape of testis specific histone H2B variant and its influence on sperm function
Source: Clin Epigenetics. 2021 May 1;13:101. doi: 10.1186/s13148-021-01088-4 (PMC8088685; doi:10.1186/s13148-021-01088-4)
Supplement: Supplementary file 3 — Additional file 3. Location of TH2B enriched regions from TSS (S1); Specificity of TH2B antibody and MNase digestion of sperm DNA (S2). [file 13148_2021_1088_MOESM3_ESM.pdf]

FIGURE S1

Number of enriched regions observed Upstream and Downstream to TSS

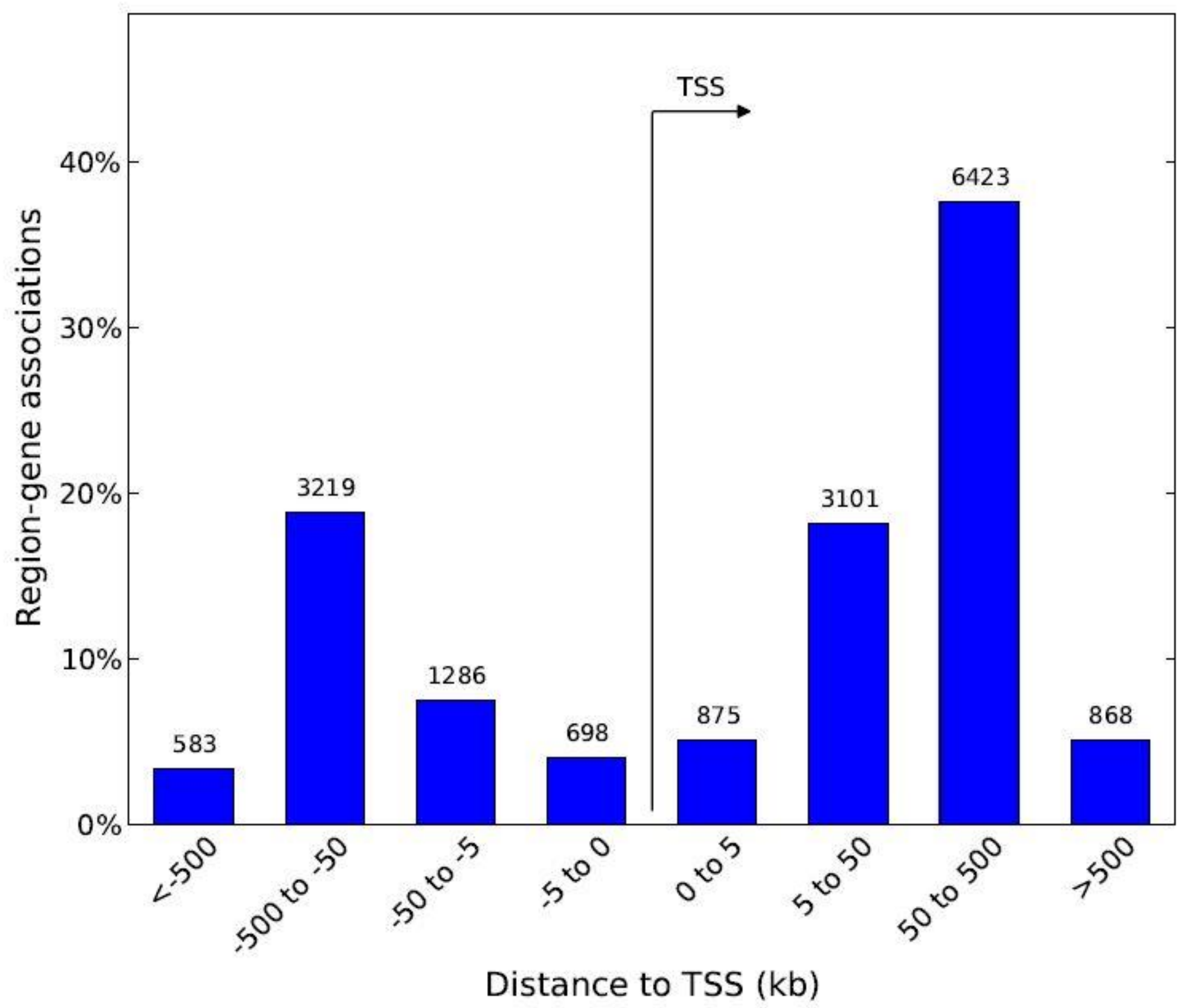

FIGURE S2

Chromatin Immunoprecipitation of TH2B from sperm

A)

Identity- 85% Similarity- 90%

```
TH2B    1 MPEVSSKGATISKKGFKKAVVKTQKKEGKKRKRTRKESYSIYIYKVLKQV
          |||  .:|.|...|||.||||.|.|||:||||||:||||||||:|||||||
H2B     1 MPE-PAKSAPAPKKGSKKAVTKAQKKDGKKRKRSRKESYSIYVYKVLKQV

TH2B   51 HPDTGISSKAMSIMNSFVTDIFERIASEASRLAHYSKRSTISSREIQTAV
          |||||||||||.||||||.|||||||.||||||||:|||||:||||||||
H2B    50 HPDTGISSKAMGIMNSFVNDIFERIAGEASRLAHYNKRSTITSREIQTAV

TH2B  101 RLLLPGELAKHAVSEGTKAVTKYTSS-     126
          |||||||||||||||||||||||||
H2B   100 RLLLPGELAKHAVSEGTKAVTKYTSSK     126
```

B)

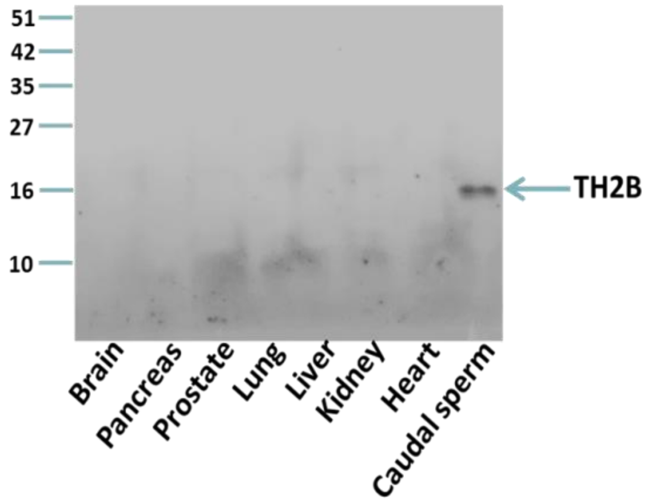

C)

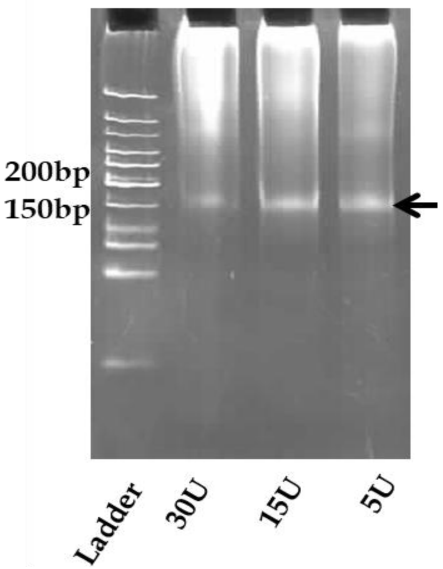

Fig.S1*: The pairwise sequence alignment of TH2B with somatic H2B. The red box represents the N terminal of proteins where both protein s differs from each other majorly (A). Western blot analysis showing TH2B present only in rat caudal sperm but not in any other somatic tissue(B). Agarose gel picture showing sperm DNA digested with different MNase concentrations. The black arrow indicates mononucleosomal DNA band (C).*
